# An Assessment of Whole Blood and Fractions by Nested PCR as a DNA Source for Diagnosing Canine Ehrlichiosis and Anaplasmosis

**DOI:** 10.1100/2012/605743

**Published:** 2012-08-22

**Authors:** Tereza Emmanuelle de Farias Rotondano, Alzira Maria Paiva de Almeida, Elane Maria Camboim Lustosa, Aline Antas Cordeiro, Expedito Kennedy Alves Camboim, Sérgio Santos de Azevedo, Paulo Paes de Andrade, Marcia Almeida de Melo

**Affiliations:** ^1^Departamento de Ciências Biológicas, Universidade Federal de Pernambuco, Avenue Professor Moraes Rego, s/n, Cidade Universitária, 50.670-901 Recife, PE, Brazil; ^2^Centro de Pesquisas Aggeu Magalhães, FIOCRUZ-PE, Avenue Professor Moraes Rego, s/n, Cidade Universitária, 50.670-901 Recife, PE, Brazil; ^3^Unidade Acadêmica de Medicina Veterinária, Universidade Federal de Campina Grande, 58.700-970 Patos, PB, Brazil; ^4^Departamento de Genética, Universidade Federal de Pernambuco, Avenue Professor Moraes Rego, s/n, Cidade Universitária, 50.670-901 Recife, PE, Brazil

## Abstract

Ehrlichiosis and anaplasmosis are tick-borne diseases. *Ehrlichia canis* and *Anaplasma platys* infect mainly white cells and platelets, respectively. The main DNA source for PCR is peripheral blood, but the potential of blood cell fractions has not been extensively investigated. This study aims at assessment of whole blood (WB) and blood fractions potential in nested PCR (nPCR) to diagnose canine ehrlichiosis and anaplasmosis. The 16S rRNA gene was amplified in 71.4, 17.8, 31.57, and 30% of the WB, granulocyte (G), mononuclear cells (M), and buffy coat (BC) samples. Compared to the WB, the sensitivity of the PCR was 42.86% for the M, and BC fractions, 21.43% for the G, and 33.33% for the blood clot (C). There was fair agreement between the WB and M, BC and C, and slight with the G. Fair agreement occurred between the nPCR and morulae in the blood smear. One animal was coinfected with *A. platys* and *E. canis*. This study provided the first evidence of *A. platys* infection in dogs in Paraíba, Brazil, and demonstrated that WB is a better DNA source than blood fractions to detect *Ehrlichia* and *Anaplasma* by nPCR, probably because of the plasma bacterial concentration following host cell lysis.

## 1. Introduction

Ehrlichiosis and anaplasmosis are important, emerging zoonotic tick-borne diseases caused by gram-negative, obligate intracellular bacteria from the Anaplasmataceae family. In the host cells, the bacteria reside in inclusion bodies (morulae), which provide a hospitable environment for survival [1, 2].

Canidae can be infected by several Anaplasmataceae agents: *Ehrlichia canis*, *E. ewingii*, *E. chaffeensis*, *Anaplasma platys*, *A. phagocytophilum*, *Neorickettsia risticii*, and *N. helminthoeca*. *Ehrlichia *and *Anaplasma* infections are transmitted through the salivary secretions of attached ticks. *Ehrlichia canis* is usually transmitted by brown dog tick (*Rhipicephalus sanguineus*) bites, which can also transmit *E. ewingii* and most likely *Anaplasma platys* [1]. The occurrence of the tick *R. sanguineus* parasitizing humans in Brazil [3] serves to warn the risk of transmission of such pathogens (*A. platys* and *E. canis*) to humans [4, 5].

*E. canis* species mainly infect monocytes, which causes canine monocytic ehrlichiosis, and *A. platys* species infect platelets, which causes canine cyclic thrombocytopenia. The *A. platys* platelet tropism is unique among ehrlichial-related organisms, even though all of these infections may result in thrombocytopenia [2]. *E. canis* is the main pathogen implicated in cases of canine ehrlichiosis in Brazil, but *A. platys* has recently been identified by PCR in samples from the South region with a prevalence ranging from 25.5% to 55% [1, 5].

The diagnosis of canine ehrlichiosis and anaplasmosis relies on the cultivation, serology, PCR, and direct microscopic examination of stained blood smears to identify intracytoplasmic morulae. Smear diagnosis has low sensitivity, as there are few bacteria present in the samples, morulae can be visualized only during the acute phase, and the percentage of infected cells is usually less than 1% [6]. Additionally, the presence of *A. platys* is cyclical, and the bacteria are easily mistaken as nonspecific inclusion bodies and staining artifacts [1, 7]. Serology is hampered by cross-reactions and cannot discriminate between a current infection and previous exposure to the pathogen. Moreover, antibody titers tend to persist for several months to years after treatment, making serology an unreliable tool for posttreatment diagnosis [8].

The first PCR-based diagnostic method for ehrlichiosis amplified the 16S rRNA gene and was reported by Iqbal et al. in 1994 [9]. Further improvements and the use of other target genes increased the sensitivity of the tests. The p30-based nested PCR (nPCR) assay has been shown to be more sensitive than the 16S rRNA-based nPCR assay [10], possibly because *E. canis* contains multiple copies of the p30 gene but only one copy of the 16S rRNA gene [11]. As opposed to single-step PCR, nPCR amplification of the 16S rRNA gene has been used more often to detect *E. canis* and *A. platys*. In both single step PCR and nPCR, the peripheral blood is frequently used as a DNA source [1, 5, 12]. Only a single report has described the use of mononuclear cells as a DNA source [9].

There is a high prevalence of canine ehrlichiosis, but there are few reports on the identification of the infectious agents; therefore, a practical diagnostic technique that can be routinely used in veterinary medicine must be established. The nPCR assay may fulfill this requirement, but the blood fraction that serves as the best DNA source must be determined beforehand. The aim of the present study was to compare the effectiveness of whole blood (WB) and blood fractions—buffy coat (BC), granulocytes (G), mononuclear fraction (M) and blood clot (C)—by nPCR to diagnose canine ehrlichiosis and anaplasmosis.

## 2. Methods

### 2.1. Samples and Cell Fractionation

Blood was collected from 21 dogs bearing suggestive clinical signs of either ehrlichiosis or anaplasmosis (petechia, ecchymosis, fever, and anorexia) and harboring ticks. Some animals also had intracytoplasmic morulae, as indicated by direct examination of blood smears and/or hematological parameters suggestive of ehrlichiosis and anaplasmosis. The dogs were selected from the veterinary hospital Universidade Federal de Campina Grande (UFCG), the Veterinary Medical Center Dr. Leonardo Torres at Patos, State of Paraiba, and at the Veterinary Hospital at Universidade Federal Rural de Pernambuco (UFRPE), at Recife, State of Pernambuco.

### 2.2. Hematology, Direct Examination of Blood Smears and Cell Fractionation

Routine platelet counts, packed cell volume, and other hematology parameters were performed at the hospitals referred to above. The reference values were those described in Jain (1993) [15]. WB smears were stained with a hematoxylin-eosin-based rapid stain (Panótico rápido, Laborclin, Brazil) and observed by microscopy (100X objective, under immersion oil). The M- and G-enriched samples were obtained from 4 mL of WB with the SepCell kit (LGC Biotecnologia, Brazil), according to the manufacturer's instructions. The BC fraction was collected from 1 mL blood that was centrifuged at 12,000 g for 10 min.

### 2.3. DNA Extraction

From each dog, a sample of blood was collected, and the DNA was extracted. Four milliliters of blood were extracted with sodium citrate and 1 mL without sodium citrate. The DNA samples from the WB (200 *μ*L), BC (50 *μ*L), M (50 *μ*L), G (100 *μ*L), and C (50 *μ*L) fractions were extracted with a commercial kit (Invisorb Spin Blood Midi kit; INVITEK), following the manufacturer's instructions. The DNA from 21 WB, 19 G and 19 M, 20 BC, and 15 C samples was used in the nPCR to amplify the *E. canis* and *A. platys* 16S rRNA sequences.

### 2.4. Nested PCR (nPCR)

The first round of PCR used 0.5 to 1.0 *μ*g of the genomic DNA, and the primers ECC and ECB were designed to amplify a 478 base-pair (bp) fragment of the *Ehrlichia* 16S rRNA [13]. The second round of PCR used a 1.0 *μ*L aliquot of the first reaction as a template and the EHCA sense/EHCA antisense [14] and EHPL sense/EHPL antisense (João Pessoa Araújo Jr.: pers. comm., 2010) primers, which were designed to amplify a 389 bp fragment for *E. canis* and 384 bp fragment for *A. platys*, respectively. Separate reactions were used to detect each species individually. The primers are described in Table 1. The primer design was confirmed with the software Primer 3 (http://fokker.wi.mit.edu/primer3/input.htm). The reaction mix contained 1X reaction buffer (50 mM KCl, 20 mM Tris-HCl (pH 8.4), and 0.1% Triton X-100), 1.75 mM MgCl_2_, 0.2 mM dNTP mix, 1 *μ*M PCR primers, 0.625 U Taq DNA polymerase, and autoclaved ultrapure water to a final volume of 25 *μ*L. The thermocycle was as follows: 94°C for 10 minutes followed by 40 cycles at 94°C for 60 seconds, 60°C for 60 seconds, 72°C for 60 seconds, and a final step of 72°C for 4 minutes before holding at 4°C. Ultra-pure autoclaved water was used as negative control in each PCR batch. The genomic DNA from confirmed *E. canis* and *A. platys* cases was used as positive controls for the *E. canis *16S rRNA and *A. platys *16S rRNA genes, respectively. Ten microliters of the final products were electrophoresed at 90 volts for approximately 1 hour in 1.5% agarose gels containing ethidium bromide in Tris-Borate EDTA (TBE). The *E. canis* and *A. platys* reactions were positive when a 389 or a 384 bp fragment was detected, respectively.

### 2.5. Statistical Analysis

The kappa and related indices were calculated by Dag Stat software [16] to determine the agreement between the results for the WB (gold standard) and blood fractions. The McNemar test was used to evaluate the concordance among DNA sources, and the Fisher's exact test was used to determine the association between thrombocytopenia, anemia, and a positive WB nPCR. The significance level was 5% for all of the analyses.

### 2.6. Ethical Considerations

The animals were used according to the guidelines of Oswaldo Cruz Foundation from Brazil's Ministry of Health.

## 3. Results

Table 2 shows the results of hematological, blood smear (direct examination), and nPCR on the WB, G, M, BC, and C samples from 21 dogs exhibiting clinical signs of ehrlichiosis. From each group, negative samples were detected. In seven animals (46.6%), identification at species level failed, as there was no amplification in the second PCR. Among them, the blood smears of five dogs were positive by direct examination and two displayed cytoplasmic inclusions.

Seven dogs (33.3%) were positive by nPCR and direct examination of blood smears (presence of morulae); inclusions within platelets were found in two blood smears. Out of the 14 blood smear-negative animals, eight (63.6%) had at least one blood fraction positive for *Ehrlichia* or *Anaplasma *by nPCR, corresponding to 57.1% false negatives by direct examination. The WB DNA samples from 66.6% (6/9) thrombocytopenic and 42.85% (3/7) anemic animals were positive by nPCR.

**Figure 1:**
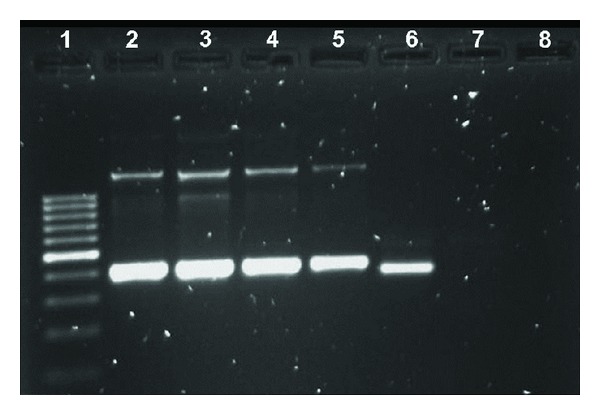
Detection of *Ehrlichia canis* in nPCR with EHCA sense and antisense primers for rRNA 16S gene. Lane 1: 100 base pair (bp) DNA ladder; lanes from 2 to 5: nPCR with DNA from WB; lane 6: *E. canis*-positive control and DNA from WB; lane 7: negative control; lane 8: nPCR-negative control.

Among 21 WB samples, 26.6% (6/21) were negative by nPCR, and 71.4% (15/21) were positive: 46.4% (7/15) for *E. canis *(Figure 1) and 6.6% (1/15) for *A. platys*. *E. canis* was identified in G samples from 1.8% (3/19), in M samples from 31.6% (6/19), and in BC samples from 31.6% (6/19) animals. One BC sample was coinfected with *E. canis *and *A. platys*. Among the C samples, 7.14% (1/14) were positive for *E. canis *and 14.3% (2/14) for *A. platys*.

Among the nPCR assays carried out in all samples (WB, G, M, BC, and C) from 11 animals, at least 63.3% (7/11) were positive; WB and C samples were simultaneously positive in 9% (1/11) and WB, M, and BC in 18.1% (2/11).

The nPCR sensitivity was 42.86% when the WB was compared to the M and BC fractions (McNemar test: *X* ^2^ = 6.13; *P* = 0.013), 21.43% compared to the G fraction (McNemar test: *X* ^2^ = 9.09; *P* = 0.003), and 33.33% compared to the C fraction (McNemar test: *X* ^2^ = 4.17; *P* = 0.041). The kappa value showed fair agreement among WB and M (Kappa = 0.28), BC (Kappa = 0.31), and C fractions (Kappa = 0.26) and slight agreement with G fraction (Kappa = 0.13). There was also fair agreement between the presence of morulae and the nPCR results (Kappa = 0.33; McNemar test: *X* ^2^ = 6.13; *P* = 0.0133).

## 4. Discussion

The direct examination of stained blood smears to detect *Ehrlichia* in dogs has a low sensitivity rate (3 to 9%). In fact, *E. canis* morulae are difficult to detect in blood smears because this organism is usually present in very low concentrations [6]. In contrast, PCR has proven to be more sensitive for detecting *Ehrlichia*; for a 16S rRNA-based PCR assay is able to detect *E. canis* DNA from a rickettsemia, which is equivalent to one infected monocyte in 10^36^ cells [1, 5, 12]. In addition to the large sensitivity differences inherent to the techniques, genotypic variants have been reported for *E. ruminantium,* and *A. platys* infects a wide range of host cells [1, 2, 17].

As expected, our study demonstrates that nPCR is more sensitive for detecting *Ehrlichia* than the direct examination of stained blood smears of dogs with suggestive clinical signs. Our results show that a 50% false negative rate may occur when only direct examination is used for diagnosis. In contrast, all animals with morulae in the blood smears were positive by nPCR for at least one of the WB or fraction samples.

The nPCR was able to detect *Ehrlichia* or *Anaplasma* DNA in 71% of the samples from dogs with suggestive clinical signs. This rate is slightly higher than that registered elsewhere in Brazil [1, 5, 12]. As previously reported [1, 5], *E. canis* (46.6%) positivity in WB was higher than for *A. platys* (6.6%).

In seven (46.6%) of the samples, there was no amplification in the second PCR, and the positives were recorded as *Ehrlichia *spp. As the primers used were specific for *E. canis* and *A. platys*, the presence of other Rickettsiales, such as* A. phagocytophilum*, *E. chaffeensis*, and *E. ewingii*, should not be disregarded because they can also form cytoplasmic inclusions [18, 19]. Furthermore *E. ewingii *was already reported in dogs in Brazil [20].

Coinfection with *E. canis* and *A. platys* was observed in an animal with a positive blood smear and that was positive for *E. canis *in the WB sample by nPCR. Cytoplasmic inclusions in the platelets were not observed, possibly due to low *A. platys* load [7]. It is worth mentioning that this is the first evidence for the involvement of *A. platys* in canine anaplasmosis in the State of Paraiba, Brazil.

The blood fraction samples that were positive for *A. platys* by nPCR were WB and C (dog no. 14) and B and C (dog no. 12). Despite the small sample size, the results suggest an increased likelihood of finding *A. platys* DNA in the BC fraction, which is more enriched with platelets than the other samples.

Contrary to previous reports [21, 22], we found that there was no statistical association between thrombocytopenia (*P* = 0.596), anemia (*P* = 0.299), and the WB nPCR results. Similar to a previous report [1], anemia occurred in only 26.6% cases. These results demonstrate that thrombocytopenia is not sufficient to diagnose either canine ehrlichiosis or anaplasmosis. Santos et al. [22] also observed a high incidence of *E. canis *infection among nonthrombocytopenic dogs. In contrast, other diseases including immune-mediated thrombocytopenia, neoplasia, inflammatory diseases, or other infectious agents can provoke thrombocytopenia [23]. The differences in prevalence may reflect the diversity in strain pathogenicity or a selection bias because thrombocytopenic dogs are more likely to be tested for ehrlichiosis.

Peripheral blood has been the main source of *Ehrlichia* DNA for PCR assays because collection of this sample is less invasive than spleen and bone marrow collection. The use of serum samples for nPCR to detect *E. canis* has been suggested previously [24]. Our results support that whole blood is the best source for *Ehrlichia* DNA in PCR assays. Indeed, the Kappa value indicates a weak correlation between nPCR results from the WB samples and those obtained with the G, M, BC, or C samples; the PCR sensitivity from the M and B samples was only 42.9%. Therefore, our data and the literature support the use of WB as the best choice for DNA source for PCR *Ehrlichia* spp. detection.

This is the first assessment of the use of different blood cell fractions as DNA sources to diagnose canine ehrlichiosis and anaplasmosis by PCR. Although the pathogens only infect leukocytes and platelets, WB is a better DNA source than any of the cellular* Ehrlichia*-enriched host cell fractions. A possible explanation may be based on the assumption that WB samples contain not only intracellular *Ehrlichia* but also organisms released by host cell lysis that are not found in the fractions. In support of this hypothesis, the 16S rRNA gene was successfully amplified by Mylonakis et al. [25] by nPCR in sera samples from naturally infected dogs. Hence, these authors recommend serum-based PCR analysis for the early diagnosis of CME when WB samples are not available. Furthermore, it was demonstrated that *E. chaffeensis* reached concentrations of ~10^8^ bacteria/mL in the plasma of SCID mice two weeks after infection [26]. There are no similar studies for *E. canis* or *A. platys*, but it is reasonable to assume that a similar scenario occurs in dogs infected with these pathogens, especially in the acute phase of the disease, when symptoms are severe, and platelet counts are usually reduced.

In conclusion, the present study demonstrates that canine WB is better than other cellular blood fractions as a DNA source to detect *Ehrlichia* and *Anaplasma* by PCR, most likely because of the bacterial concentration in the plasma following host cell lysis.

## Figures and Tables

**Table 1:** The primer sequences for the 16S rRNA gene used to detect the *E. canis* and *A. platys* by the nPCR reactions.

Primer	Etiological	Primer sequences	Reference	Expected amplified	From-to
identification	agent			segment length	(bp)
ECC	*E*. spp.	AGAACGAACGCTGGCGGCAAGCC	Dawson et al. [13]	478 bp	13–490
ECB	*E*. spp.	CGTATTACCGCGGCTGCTGGC			
EHCA sense	*E. canis*	CAATTATTTATAGCCTCTGGCTATAGC	Wen et al. [14]	389 bp	58–446
EHCA antisense	*E. canis*	TATAGGTACCGTCATTATCTTCCCTAT			
EHPL sense	*A. platys*	TTTTTGTCGTAGCTTGCTATGATA	João Pessoa Araújo Jr.,	384 bp	49–432
EHPL antisense	*A. platys*	TGTGGGTACCGTCATTATCTTCCCCA	pers. comm		

**Table 2:** Hematological, blood smear direct examination and whole blood (WB), granulocytes (G), peripheral blood mononuclear cells (M), buffy coat (BC) and blood clot (C) PCR results of dogs with clinical signs of ehrlichiosis.

Animal ID	Packed cell volume^∗∗^	Leukocytes^∗∗∗^	Platelets^∗∗∗∗^	Blood smear	PCR				
					WB	G	M	BC	C
01	37	18,100	314,000	Positive	*Ehrlichia *spp*. *	Negative	Negative	Negative	∗
02	45	6,200	49,000	Negative	*E. canis*	*E. canis*	*E. canis*	*E. canis*	∗
03	51	8,000	195,000	Negative	*Ehrlichia *spp*. *	Negative	Negative	Negative	Negative
04	∗	∗	∗	Negative	*E. canis*	*E. canis*	*E. canis*	*E. canis*	∗
05	27	35,300	334,000	Negative	Negative	∗	∗	Negative	Negative
06	46	8,200	257,000	Negative	Negative	Negative	Negative	Negative	∗
07	51	6,200	199,000	Negative	*Ehrlichia *spp*. *	Negative	Negative	Negative	∗
08	37	9,700	248,000	Negative	Negative	Negative	Negative	Negative	Negative
09	51	20,250	595,000	Positive	*Ehrlichia *spp*. *	Negative	Negative	Negative	∗
10	∗	∗	∗	Negative	*E. canis*	Negative	*E. canis*	∗	*E. canis*
11	16	65,100	67,000	Negative	*E. canis*	*E. canis*	*E. canis*	*E. canis*	∗
12	∗	∗	∗	Positive	*E. canis*	∗	∗	*E. canis/A. platys*	*A. platys*
13	∗	∗	∗	Positive	*E. canis*	Negative	*E. canis*	*E. canis*	Negative
14	41	∗	119,000	Negative	*A. platys*	Negative	Negative	Negative	*A. platys*
15	21	12,900	116,000	Negative	Negative	Negative	Negative	Negative	Negative
16	27	14,800	148,000	Positive	*Ehrlichia *spp*. *	Negative	Negative	Negative	Negative
17	35	10,000	∗	Negative	Negative	Negative	Negative	Negative	Negative
18	31	—	44,400	Negative	Negative	Negative	Negative	Negative	Negative
19	31	27,100	408,000	Positive	*Ehrlichia *spp*. *	Negative	Negative	Negative	Negative
20	41	13,100	277,920	Positive	*Ehrlichia *spp*. *	Negative	Negative	Negative	Negative
21	42	21,900	21,900	Negative	*E. canis*	Negative	*E. canis*	*E. canis*	Negative

ID: Identification; RV: reference value (Jain, [15]); ^∗^not performed; ^∗∗^Packed cell volume (RV: 37–55%); ^∗∗∗^Leukocytes (×10^3^/*μ*L; RV: 6–17); ^∗∗∗∗^Platelets (×10^5^/*μ*L; RV: 2–5).
